# Novel Microorganisms Contribute to Biosulfidogenesis in the Deep Layer of an Acidic Pit Lake

**DOI:** 10.3389/fbioe.2022.867321

**Published:** 2022-07-13

**Authors:** Diana Ayala-Muñoz, William D. Burgos, Javier Sánchez-España, Carmen Falagán, Estelle Couradeau, Jennifer L. Macalady

**Affiliations:** ^1^ Department of Civil and Environmental Engineering, The Pennsylvania State University, University Park, PA, United States; ^2^ Centro Nacional Instituto Geológico Minero de España (IGME), CSIC, Madrid, Spain; ^3^ School of Biological Sciences, University of Portsmouth, Portsmouth, United Kingdom; ^4^ Department of Ecosystem Science and Management, The Pennsylvania State University, University Park, PA, United States; ^5^ Department of Geosciences, The Pennsylvania State University, University Park, PA, United States

**Keywords:** biosulfidogenesis, acidic pit lake, metagenomics, metatranscriptomics, metagenome-assembled genomes (MAGs), acidophiles, microbial sulfate reduction (MSR)

## Abstract

Cueva de la Mora is a permanently stratified acidic pit lake with extremely high concentrations of heavy metals at depth. In order to evaluate the potential for *in situ* sulfide production, we characterized the microbial community in the deep layer using metagenomics and metatranscriptomics. We retrieved 18 high quality metagenome-assembled genomes (MAGs) representing the most abundant populations. None of the MAGs were closely related to either cultured or non-cultured organisms from the Genome Taxonomy or NCBI databases (none with average nucleotide identity >95%). Despite oxygen concentrations that are consistently below detection in the deep layer, some archaeal and bacterial MAGs mapped transcripts of genes for sulfide oxidation coupled with oxygen reduction. Among these microaerophilic sulfide oxidizers, mixotrophic *Thermoplasmatales* archaea were the most numerous and represented 24% of the total community. Populations associated with the highest predicted *in situ* activity for sulfate reduction were affiliated with *Actinobacteria, Chloroflexi,* and *Nitrospirae* phyla, and together represented about 9% of the total community. These MAGs, in addition to a less abundant *Proteobacteria* MAG in the genus *Desulfomonile,* contained transcripts of genes in the Wood-Ljungdahl pathway. All MAGs had significant genetic potential for organic carbon oxidation. Our results indicate that novel acidophiles are contributing to biosulfidogenesis in the deep layer of Cueva de la Mora, and that *in situ* sulfide production is limited by organic carbon availability and sulfur oxidation.

## Introduction

Biosulfidogenesis is a microbial process applied in environmental management and industry. Biosulfidogenesis implies the microbial reduction of oxidized sulfur compounds (e.g., sulfate and elemental sulfur) to sulfide. It is especially beneficial for remediation of acidic rock drainage systems, which are usually characterized by high metal concentrations. Sulfide reacts with metal cations (e.g., Zn, Co., Cu, Ni and Cd) to form highly insoluble metal sulfides which can then be sequestered or recovered (Ňancucheo and Johnson, 2012; Johnson et al., 2020). At pH below 5, biosulfidogenesis can also contribute to acid neutralization through the consumption of protons during processes such as sulfate reduction (Johnson et al., 2019).

Few sulfide-producing acidophiles have been isolated or otherwise characterized. The bacterial strains isolated to date belong to the phylum *Firmicutes* and are acid-tolerant rather than truly acidophilic (Johnson et al., 2020)*.* For example, *Thermodesulfobium narugense,* isolated from a hot spring in Japan, grows between pH 4 and 6.5 (Mori et al., 2003). A *Desulfosporosinus* species (GBSRB4.2) isolated from sediments collected at a coal mine site in the United States grows between pH 4 and 6.3 (Senko et al., 2009). *Desulfosporosinus acidiphilus,* isolated from acid rock drainage in France, grows between pH 3.6 and 5.5 (Alazard et al., 2010). *Desulfosporosinus acididurans,* isolated from sediments of the river Tinto (Spain), grows between pH 3.8 and 7 (Sánchez-Andrea et al., 2015). *Thermodesulfobium acidiphilum,* isolated from a thermal site in Russia, grows between pH 3.7 and 6.5 (Frolov et al., 2017). *Desulfosporosinus* sp. I2, isolated from gold mine tailings in Siberia, grows between pH 1.7 to 7.0 (Mardanov et al., 2016). *Desulfosporosinus metallidurans*, isolated from a microbial mat in a tailing dam at a gold ore mining site in Russia, grows between pH 4.0 to 7.0 (Panova et al., 2021)**.** Many of these strains can also reduce elemental sulfur to sulfide. Other acidophiles in laboratory culture are capable of elemental sulfur reduction but not sulfate reduction. For example, the bacterium *Desulfurella amilsii* and the archaea *Thermoplasma acidophilum, T. volcanium*, *Acidianus brierleyi, A. infernus, Sulfurisphaera ohwakuensis,* and *Stygiolobus azoricus* reduce elemental sulfur to sulfide under acidic conditions (Segerer et al., 1986; Segerer et al., 1988; Segerer et al., 1991; Kurosawa et al., 1998; Florentino et al., 2017). These isolates have been obtained mainly from sediment enrichments.

No biosulfidogenic microorganisms have been isolated from the water column of acidic environments to date. However, molecular analyses have identified populations related to sulfate and elemental sulfur reducers. For example, the deep layers of the Brunita and Filón Centro acidic pit lakes in Spain have been reported to be dominated by 16S rRNA gene sequences related to the genus *Desulfomonile* (Sánchez-España et al., 2020a; Van der Graaf et al., 2020). In another IPB acidic pit lake, La Zarza, elemental sulfur-reducing archaea related to *Acidianus* and *Thermoplasma* spp. as well as sulfur-disproportionating bacteria related to *Desulfocapsa* spp. were detected in the deep layer of the water column (Van der Graaf et al., 2020). Microorganisms affiliated with the sulfate-reducing genus *Desulfomonile* were reported in the chemocline of the IPB pit lake Cueva de la Mora (Falagán et al., 2014). The discovery of these poorly studied, sulfide-producing populations has led to renewed interest in the idea that they could promote *in-situ* metal sulfide precipitation, thus improving water quality.

Cueva de la Mora (CM) is a permanently stratified acidic pit lake located near Huelva, Spain. It has undergone more than 40 years of hydrogeochemical evolution (Sánchez-España et al., 2009) and has been extensively studied over the past decade. It has stable vertical geochemical gradients with depth, and the pH is ∼2 in the upper layer, ∼3 in the chemocline, and ∼4 in the deep layer (Sánchez-España et al., 2009). The lake has levels of nutrients sufficient to support the abundant growth of algae in the photic zone (Wendt-Potthoff et al., 2012; Sánchez-España et al., 2020b). Phytoplankton in the relatively shallow photic zone produce labile organic carbon that fuels processes such as sulfate reduction in the chemocline (Diez-Ercilla et al., 2014). The deep layer of CM has the highest concentrations of sulfate (126 mM), metal (loid)s (e.g., 1.7 mM of Zinc, 0.23 mM A), and carbon dioxide (29 mM) compared to the upper layers (26–41 mM of sulfate, 0.19–0.53 mM of Zn, and 1.3–7 mM of CO_2_) (Sánchez-España et al., 2009; Sánchez-España et al., 2020b).

Given the non-detection of oxygen in the deep layer (<0.02 mg/L O_2_), we expected to find active microbial sulfate reduction. Previous microbiological studies of CM attempted to detect dissimilatory sulfur cycling through culture-dependent techniques (Wendt-Potthoff et al., 2012) or terminal restriction enzyme fragment length polymorphism (T-RFLP) analysis of 16S rRNA amplicons (Falagán et al., 2014). These studies reported low numbers of sulfate reducing populations and no detectable sulfate reduction activity in the deep layer of CM. Attempts to enrich sulfate reducing strains from the deep layer were not successful (Falagán et al., 2014). In a recent study, CM microbial communities and their biogeochemical roles were better resolved using metagenomics and metatranscriptomics (Ayala-Muñoz et al., under revision). In this earlier work, we predicted that many deep layer populations could contribute to sulfur cycling. In the current study we focused our analysis on assembled genomes representing populations potentially involved in biosulfidogenesis. The specific objectives of this work were to: 1) reconstruct metagenome-assembled genomes (MAGs) representing the most abundant and active phyla in the deep layer of the lake, 2) identify genes and transcripts involved mainly in carbon, sulfur cycling in each MAG, and 3) describe the metabolic capabilities of the populations that contribute the most to biosulfidogenesis in the deep layer of the lake.

## Methods

### Sample Collection, DNA/RNA Extraction, and Sequencing

Details of the sampling campaign, DNA/RNA extraction, and sequencing were previously described (Ayala-Muñoz et al., 2020). Briefly, 5 L of water were collected in triplicate at CM at 35 m depth using a Van Dorn limnological ‘horizontal’ sampling bottle (KC Denmark A/S, Silkeborg, Denmark). For each replicate water sample, 3 L were used for collection of microbial biomass for DNA extractions, and 1 L was filtered for RNA extractions. Water was prefiltered through a 2 µm pore size glass fiber filter to reduce clogging, followed by a 0.2 µm pore size polyethersulfone (PES) sterivex filter. As in previous studies, the data pertained to planktonic cells and particles retained on a 0.2 µm filter but not retained on a 2 µm filter (Ayala-Muñoz et al., 2020; Ayala-Muñoz et al., 2022). This protocol was necessary to exclude abundant mineral particles that clogged 0.2 µm filters before sufficient microbial biomass could be obtained. We are aware that there might be microbial populations associated with mineral precipitates or debris bigger than 2 μm, or microbial populations smaller than 0.2 µm, but we expect most of the biomass to fall within 0.2–2 microns size range, a follow-up study will be necessary to characterize microbial populations outside this range.

Sterivex filters were placed immediately on ice and cryo-shipped to the Pennsylvania State University, Pennsylvania, United States, where they were stored at -80C until DNA/RNA extraction. Extractions were conducted with Qiagen DNAeasy Powerwater Kit for DNA and Qiagen RNAeasy PowerMicrobiome Kit (Qiagen, Venlo, Netherlands) for RNA. Quality and quantity of the extracts was verified using Qubit^®^ 2.0 Fluorometer (Invitrogen, Carlsbad, CA, United States) and Bioanalyzer 2100 RNA 6000 Pico Assay (Agilent, Santa Clara, CA, United States). Only the best two replicates were used for nucleic acid sequencing. Metagenome library preparation was performed using Illumina’s NexteraXT library preparation kit (Illumina, San Diego, CA, United States). Double stranded cDNA synthesis and metatranscriptome library preparation was performed using the Tecan RNA Trio library preparation kit (Tecan, Mannedorf, Switzerland). Sequencing was conducted on an Illumina Hiseq 4000 platform (Illumina, San Diego, CA, United States) using 150 bp paired end chemistry.

### Processing of Metagenomes and Metatranscriptomes

Details of the processing of metagenomes and metatranscriptomes were also previously described (Ayala-Muñoz et al., 2020). Two metagenomic and two metatranscriptomic datasets were obtained. After quality-filtering and trimming, metagenomic short-reads were *de-novo* assembled with Megahit v1.1.2 (Li et al., 2016). We assembled the two replicate metagenomes separately and also produced a co-assembly. The metatranscriptomes were subjected to *in silico* removal of ribosomal RNA sequences using sortmeRNA v2.1 (Kopylova et al., 2012). The remaining RNA reads (mostly mRNAs) were mapped to the metagenomic co-assembly with BBMap (JGI, 2017) (26) (JGI, 2017) (26) (JGI, 2017) (26) (JGI, 2017) (26) (JGI, 2017) (26) (Kopylova et al., 2012) (25) (Kopylova et al., 2012) (24) (Ayala-Muñoz et al., 2022) (min_id = 0.95 and slow mode). Approximately 50% of the mRNA reads from only one of the two metatranscriptomes (CM35_1) mapped to the respective metagenome contigs. The other metatranscriptomic dataset (CM35_2) with less than 1% of mapped mRNA reads to its respective metagenomic co-assembly was not used for downstream analysis. We speculate that the metatranscriptome from CM35-2 failed to map to its corresponding metagenome because of a contamination issue.

### Reconstruction of Metagenome-Assembled Genomes (MAGs)

We applied a combination of bioinformatic approaches to reconstruct metagenome-assembled genomes (MAGs) from metagenomes. Two *de-novo* individual assemblies (one per replicate) and one co-assembly were obtained using Megahit v1.1.2 with default parameters (Li et al., 2016). A subset of randomly selected reads representing 100% coverage of the most abundant taxa in each metagenome was also assembled with metaSPAdes employing the default parameters (Nurk et al., 2017). The latter approach was conducted to maximize the binning of MAGs from the most abundant taxa (i.e., *Thermoplasmatales*). We mapped reads to each assembly with Bowtie v2.2.4 using default parameters (Langmead et al., 2009). Maxbin 2.0 (-min_contig_length = 1500, -prob_threshold 0.95, and -markerset 40 for archaeal dominated samples) (Wu et al., 2016) and MetaBat v2.12.1 (Kang et al., 2019) were used to bin the two individual assemblies, the co-assembly, and the read-subset assemblies. A total of 361 MAGs were obtained using this branched pipeline. DASTool (Sieber et al., 2018) and dRep v2.3.2 (Olm et al., 2017) with default options were used to select the best non-redundant MAGs (a total of 43). CheckM was used to analyze the quality of the MAGs. Finally, we used FastAni v1.3 (Jain et al., 2018) to select MAGs with an average nucleotide identity (ANI) of <96.5% as representatives of potentially different species. MAGs with ANI >96.5% were grouped together and only the best quality MAG from each group was further analyzed (Supplementary Table S1), resulting in a total of 18 MAGs (Supplementary Table S2).

### Curation, Phylogenetic Analysis and Population Relative Abundances

Taxonomic annotation of the 18 MAGs was conducted with GTDB-Tk v1.3 (Genome Taxonomy Database) (Chaumeil et al., 2019) and the microbial genome atlas webserver (MiGA, NCBI-Prok Database) (Rodriguez-R et al., 2018). In parallel, genes from each MAG were predicted with Prodigal v2.6.3 (Hyatt et al., 2010). Genes and contigs from each MAG were taxonomically annotated with the bins annotation tool (BAT), using the CAT_prepare_20200618 database files and flag --top 11 (von Meijenfeldt et al., 2019). Contigs from a MAG corresponding to a different phylum from that assigned by GTDB-Tk were manually removed prior to downstream analyses. CheckM and GTDB-Tk were run again over the curated MAGs. A multiple sequence alignment obtained from GTDB-Tk and based on 122 archaeal- and 120 bacterial-specific marker proteins was used to construct a concatenated phylogenetic tree. Maximum likelihood (ML) phylogenies for archaeal and bacterial genomes were inferred with raxML (-x 12345 -p 2352890 -# 1000 -m PROTGAMMAAUTO) (Stamatakis, 2014). The tree was customized with Itol (Letunic and Bork, 2016). DNA reads were mapped to the contigs of each MAG using BBmap.sh (min_id = 0.95, slow mode), and relative abundances of taxa were calculated based on total DNA reads mapped to the metagenomic coassemblies.

### Metabolic Potential and Predicted Activity

The curated versions of the 18 MAGs were functionally annotated with METABOLIC v4.0 (Zhou et al., 2020). This bioinformatic pipeline predicts genes with Prodigal V2.6.3. Annotation was conducted with hmmsearch (HMMER 3.1b2) (Eddy, 2011) using databases including KOFAM (Aramaki et al., 2019), TIGRFAM (Haft et al., 2003), Pfam (Finn et al., 2014), CAZy (Lombard et al., 2013) and a database of 143 custom HMM profiles for genes involved in sulfur metabolism. The quality-filtered DNA reads (metagenome) and mRNA reads (metatranscriptome) from CM35_1 (replicate 1), were mapped to the predicted genes of each MAG with BBmap (min_id = 0.95, slow mode) (JGI, 2017; Miller et al., 2011) (JGI, 2017; Miller et al., 2011). TPM values summed per functional group were used to yield normalized values for gene or transcript abundance considering the relative abundance of each MAG. Normalized gene and transcript abundances were calculated as TPM values (number of DNA or mRNA reads mapped to the predicted gene (*10^6^) divided by length/sum of DNA or mRNA reads mapped to all genes divided by length per MAG). The gene and transcript profiles were converted to functional profiles by summing the normalized abundance of the annotated genes in each functional group. Plots were generated in R and customized in Inkscape. The KEGG annotations of each MAG coupled with KEGG Mapper (Kanehisa and Sato, 2020) were used for generating conceptual representations of MAGs with predicted *in-situ* activity for biosulfidogenesis. The representations were constructed in BioRender.com.

## Results

### Taxonomic Affiliations of Representative MAGs

**FIGURE 1:**
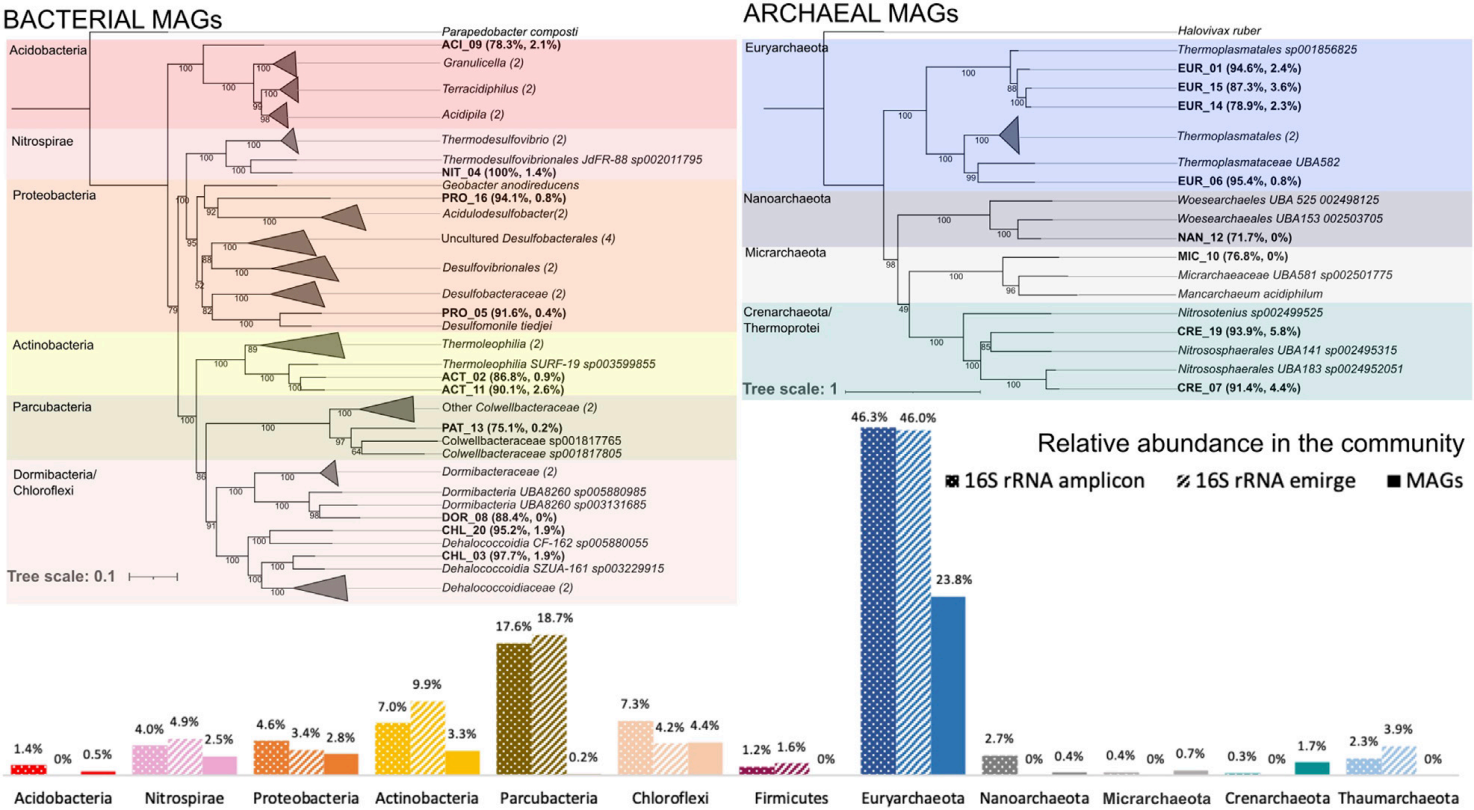
Concatenated phylogenetic trees for bacterial and archaeal MAGs recovered from the CM deep layer inferred by maximum-likelihood in raxML (Stamatakis, 2014) using multiple sequence alignments from the GTDB taxonomy analysis. Cultured and uncultured representatives of each phylum were included as references. MAG names are in bold along with the percentage of completeness and contamination. Triangles are proportional to the sequence divergence among species included in each clade. The bacterial tree is rooted by *Parapedobacter composti.* The archeal tree is rooted by *Halovivax ruber.* Along the bottom, barplots represent relative abundances of phyla in the community. In the legend, “16S rRNA amplicon” refers to the abundance of amplicon sequence variants (ASVs) relative to all ASVs in the community, “16S rRNA emirge” refers to the abundance of affiliated EMIRGE-reconstructed 16S rRNA sequences from metagenomes relative to all EMIRGE-reconstructed 16S rRNAs in the community, and “MAGs” refers to the abundance of reads mapping to a given MAG relative to all reads in the metagenome. Bar colors match the color of phyla in the trees. Values above each barplot represent relative abundances. Methods applied to obtain the “16S rRNA amplicon” and the “16S rRNA emirge” were detailed previously in Ayala-Muñoz et al., under revision). See the methods section to see how MAGs were processed.

The 18 MAGs retrieved in this study represented the majority of phyla previously described in the deep layer of Cueva de la Mora. Total relative abundances were based on 16S rRNA amplicon sequencing and 16S rRNA reconstruction from metagenomes using EMIRGE (Miller et al., 2011). The most abundant archaeal phylum (*Euryarchaeota*; 46.0% by EMIRGE, 46.3% by amplicon sequencing) was represented by four MAGs (EUR_01, 06, 14 and 15) with a total relative abundance of 23.8% (Figure 1 lower panel). The most abundant bacterial phylum (*Parcubacteria*; 18.7% by EMIRGE, 17.6% by amplicon sequencing) was represented by only one MAG (PAT_13) with a relative abundance of 0.2%. The other phyla were represented by one or two MAGs with total relative abundances comparable to what was previously described based on 16S rRNA amplicon sequencing (Ayala-Muñoz et al., 2022) and 16S rRNA reconstruction from metagenomes using EMIRGE (Ayala-Muñoz et al., 2020). DOR_08 represented the *Candidatus* Dormibacteria phylum. This phylum, formerly known as candidate division AD3, has been reported in soil or sediment samples of extreme environments including acid mine drainage sites (Mesa et al., 2017). In the Silva taxonomic database v.132 (Quast et al., 2013), AD3 was a class of the phylum *Chloroflexi,* which is how it was presented previously (Ayala-Muñoz et al., under revision). No MAGs from the *Firmicutes* or *Thaumarchaeaota* phyla were reconstructed.

None of the eighteen MAGs had high ANI (>95%) to genomes included in the Genome Database Taxonomy or the NCBI prokaryotic database (Supplementary Table S2). Eleven MAGs belonged to the *Bacteria* domain (Figure 1). ACI_09 (*Acidobacteria*) formed a separate branch from the clade that includes *Granulicella, Terracidiphilus* and *Acidipila*. NIT_04 (*Nitrospirae*) is closely affiliated with the uncultured *Thermodesulfovibrionales* JdFR-88 and separate from the clade formed by cultured *Thermodesulfovibrio* strains. PRO_16 (*Proteobacteria*) formed a clade with *Candidatus* Acidulodesulfobacter (formerly Sva0485) strains. PRO_05 clustered with *Desulfomonile tiedjie* in a clade shared with other Desulfobacteraceae. ACT_02 and ACT_11 (*Actinobacteria)* fell into a clade with *Thermoleophilia* strains. PAT_13 (*Parcubacteria*) was loosely affiliated with Colwellbacteraceae. Finally, DOR_08, CHL_20, CHL_03 are part of the phyla *Candidatus* Dormibacteria (formerly candidate division AD3 represented as a class division of *Chloroflexi* in the Silva taxonomic database v.132) and *Chloroflexi*. DOR_08 formed a clade with *Dormibacteria* strains, while CHL_20 and CHL_03 fell into a clade with *Dehalococoidia* strains. CHL_20 and CHL_03 potentially belong to different families. CHL_20 clustered with *Dehaloccoidi*a CF_162 while CHL_02 clustered with *Dehalococcoidia* SZUA-161.

Seven MAGs belonged to the *Archaea* domain (Figure 1)*.* EUR_01, 06, 14, and 15 (*Euryarchaeota*) clustered within the order *Thermoplasmatales*. EUR_01, 15 and 14 are affiliated with the uncultivated *Thermoplasmatales* sp01856825. EUR_06 is part of a different clade where cultured *Thermoplasmatales* were found. NAN_12 (*Nanoarchaeota*) is affiliated with the *Woesearchaeles*. MIC_10 (*Micharchaeota*) is affiliated with the Micharchaeaceae order. CRE_19 and CRE_07 (formerly *Crenarchaeota*, now *Thermoprotei*) are part of the *Nitrosphaerales* clade. CRE_19 is affiliated with *Nitrosospaherales* UBA141 and CRE_07 is affiliated with *Nitrososphaerales* UBA183.

### Carbon Cycling

**FIGURE 2:**
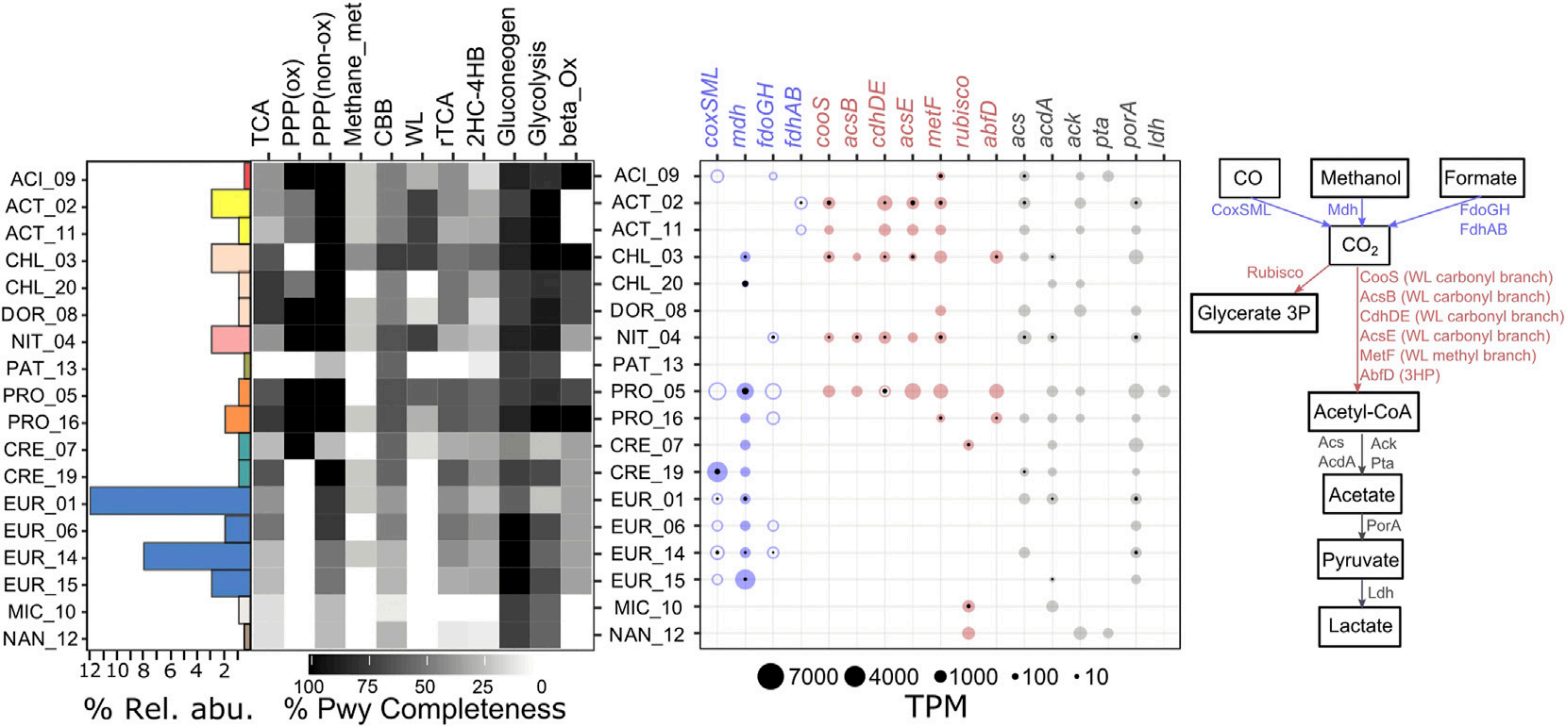
Pathways and genes involved in carbon cycling from MAGs recovered from the CM deep layer. Pathway completeness is shown at left, specific marker gene abundance is shown at center, and pathways are shown at right. MAGs are displayed in alphabetical order within each domain with bacteria on top. Bars represent abundances of individual MAGs relative to the whole metagenome (% Rel. abu.) and are shown in colors that match Figure 1. The heatmap represents the percentage of completeness of specific carbon-related pathways (% Pwy Completeness). Completeness was assessed based on the number of reactions covered by at least one enzyme in a pathway using the KEGG PathwayReconstruction web-tool. For bubble plot, gene names and bubbles are color-coded according to the pathway scheme on the right. Color-filled bubbles correspond to genes (metagenome). Black-filled bubbles correspond to transcripts (metatranscriptome). Gene complexes with missing genes are represented by un-filled bubbles. Bubble size corresponds to TPM values. KOs and further descriptions of each gene are shown in Supplementary Table S3. TCA: tricarboxylic acid cycle; PPP: pentose phosphate pathway; ox: oxidative, non-ox: non-oxidative; Methane_met: methanogenesis from acetate; CBB: Calvin-Benson-Bassham cycle; WL: Wood Ljungdahl; rTCA: reductive TCA cycle; 2HC-4HB: Dicarboxylate hydroxybutyrate cycle; beta_Ox: beta-Oxidation of fatty acids; CoxSML: aerobic carbon-monoxide dehydrogenase; Mdh: methanol dehydrogenase; FdoGH: formate dehydrogenase; FdhAB: formate dehydrogenase (coenzyme F420); CooS: anaerobic carbon-monoxide dehydrogenase catalytic subunit; AcsB; acetyl-CoA synthase; CdhDE: acetyl-CoA decarbonylase/synthase, CODH/ACS complex; AcsE: 5-methyltetrahydrofolate corrinoid/iron sulfur protein methyltransferase; MetF: methylenetetrahydrofolate reductase (NADPH); Rubisco: ribulose biphosphate carboxylase; AbdfD: 4-hydroxybutyryl-CoA dehydratase/vinylacetyl-CoA-Delta-isomerase; Acs: acetyl-CoA synthetase; AcdA: acetyl coenzyme A synthetase (ADP forming); Ack: acetate kinase; Pta: phosphate acetyltransferase; PorA: pyruvate ferredoxin oxidoreductase; Ldh: l-lactate dehydrogenase.

**FIGURE 3:**
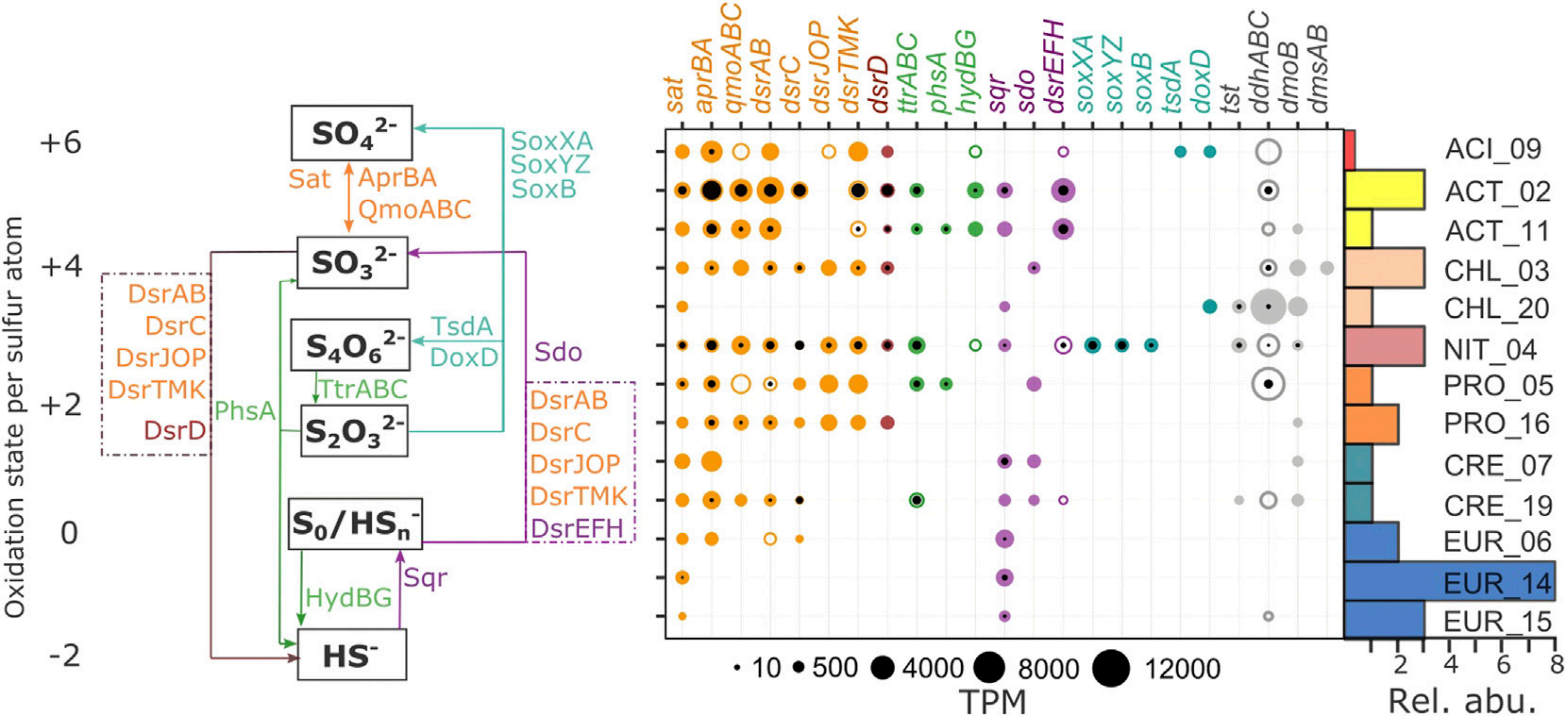
Genes involved in dissimilatory sulfur cycling found in MAGs recovered from the CM deep layer. Pathways are shown at left, specific marker gene abundances are shown at center, and relative abundances of MAGs are shown at right. Gene names and bubbles are color-coded according to the pathways at left. Color-filled bubbles correspond to genes (metagenome). Black-filled bubbles correspond to transcripts (metatranscriptome). Gene complexes with missing genes are represented by unfilled bubbles. Bubble sizes correspond to TPM values. Bars represent abundances of individual MAGs relative to the whole metagenome (% Rel. abu.) and are color-coded according to the taxonomic affiliation in Figure 1. MAGs are displayed in alphabetical order within domains with bacterial MAGs at the top. KOs and further descriptions of each gene are displayed in Supplementary Table S4. Sat: sulfate adenylyltransferase; AprBA: adenylylsulfate reductase; QmoABC: quinone-modifying oxidoreductase; DsrABCJOPTMKD: dissimilatory sulfite reductase; TtrABC: tetrathionate reductase; PhsA: thiosulfate reductase/polysulfide reductase; HydBG: sulfhydrogenase (sulfur reductase); Sqr: sulfide:quinone oxidoreductase; Sdo: sulfur dioxygenase; DsrEFH: dissimilatory sulfite reductase; SoxXA: l-cysteine S-thiosulfotransferase; SoxYZ: sulfur-oxidizing protein; SoxB: S-sulfosulfanyl-l-cysteine sulfohydrolase; TsdA: tetrathionateforming diheme cytochrome c thiosulfate dehydrogenase (reversible); DoxD: thiosulfate dehydrogenase (quinone) large subunit; Tst: thiosulfate/3-mercaptopyruvate sulfurtransferase rhodanese; DdhABC: dimethylsulfide dehydrogenase; DmoB: dimethyl-sulfide monooxygenase; DmsAB: anaerobic dimethyl sulfoxide reductase.

**FIGURE 4:**
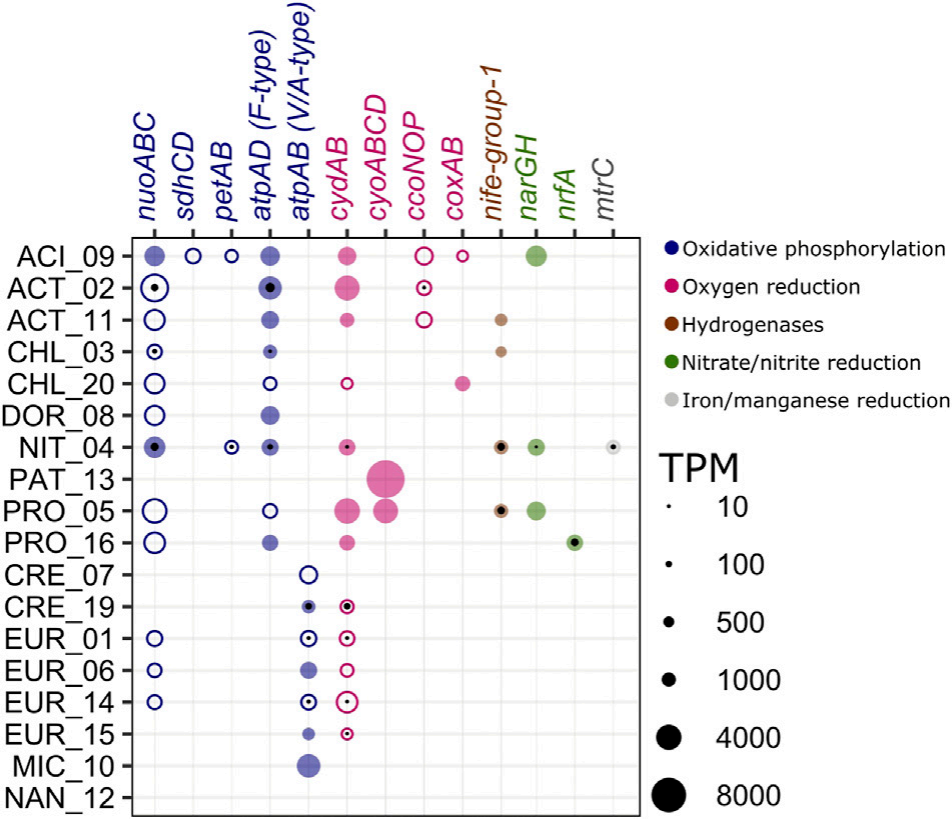
Genes involved in other respiration pathways found in MAGs recovered from the CM deep layer. MAGs are displayed in alphabetical order within domains with bacterial MAGs at the top. Gene names and bubbles are color-coded according to the pathways listed on the right. Color-filled bubbles correspond to genes (metagenome). Black-filled bubbles correspond to transcripts (metatranscriptome). Gene complexes with missing genes are represented by un-filled bubbles. Bubble sizes correspond to TPM values. KOs and further descriptions of each gene are displayed in Supplementary Table S5 nuoABC: NADH-quinone oxidoreductase; sdhCD: succinate dehydrogenase/fumarate reductase; petAB: ubiquinol-cytochrome c reductase cytochrome b/c1; atpAD (F-type): F-type H+/Na + -transporting ATPase; atpAB (V/A-type): V/A-type H+/Na + -transporting ATPase; cydAB: cytochrome bd ubiquinol oxidase; cyoABCD: cytochrome o ubiquinol oxidase; ccoNOP: cytochrome c oxidase cbb3-type; coxAB: cytochrome c oxidase; nife-group-1: Ni-Fe Hydrogenase, H2-uptake; narGH: nitrate reductase/nitrite oxidoreductase; nrfA: nitrite reductase (cytochrome c-552); mtrC: decaheme c-type cytochrome, OmcA/MtrC family.

While MAGs are *in silico* representations of environmental species populations, for brevity, we will refer to MAGs containing and expressing genes. The majority of MAGs contained and expressed genes involved in organic carbon oxidation (Figure 2, Supplementary Table S3). We have chosen to use dot plots in Figures 2–4, and Figure 7 primarily to present both the presence/absence of functional potential and gene expression, and to distinguish gene complexes with missing genes (filled vs. unfilled dots). We do not evaluate or discuss our results based on quantitative TPM values because mapping genomic and transcriptomic reads back to MAGs could produce artifacts. As far as we know, there is no standard way yet to perform this type of analysis. Based on recent studies, we decided to implement an approach like the one described by Salazar et al. (2019) (Salazar et al., 2019).

Bacterial MAGs, with the exception of PAT_13, have complete (100%) or close to complete pathways (>50%) for the tricarboxylic acid (TCA) cycle. Among the archaeal MAGs, only CRE_19 and EUR_16 had a TCA cycle with >50% completeness. With respect to the pentose phosphate pathway (PPP), most of the bacterial MAGs had almost complete PPP, oxidative (ox) and non-oxidative (non-ox) branches. Archaeal MAGs only had the PPP non-oxidative branch with CRE_19 and all EUR_MAGs having the most complete pathways. Most MAGs contained >50% complete pathways for gluconeogenesis and glycolysis. The bacterial MAGs ACI_09, CHL_03, CHL_20, DOR_08, PRO_05, PRO_16 had >50% complete pathways for beta oxidation of fatty acids.

With respect to one-carbon metabolisms, most of the MAGs lacked genes involved in acetoclastic methanogenesis or had incomplete pathways (<50%, Figure 2, Supplementary Table S3). We also searched for genes involved in carbon monoxide (CO) oxidation (*coxSML*), methanol oxidation (*mdh*), and formate oxidation (*fdo, fdh*). Strikingly, all four EUR_ MAGs and CRE_19 had genes involved in CO oxidation, some of which were actively expressed. Two bacterial MAGs (ACI_09 and PRO_05) contained at least one gene involved in CO oxidation whereas CRE_19 had all the Cox subunits (*coxSML*). ACI_09, EUR_01, and EUR_06 only had *coxS*, and EUR_14 and EUR_15 only had *coxSM*. Carbon monoxide can be a product of peroxidation of membrane lipids due to oxidative stress from metal toxicity (Wolff and Bidlack, 1976). Almost all archaeal MAGs (CRE_19 and EUR_01, 06, 14, 15), contained, and in some cases expressed, genes involved in methanol oxidation, whereas few bacterial MAGs (CHL_03, CHL_20, and PRO_05) contained those genes. Methanol can be a subproduct of dead phytoplankton sinking from the upper layers (Mincer and Aicher, 2016). Finally, with respect to formate oxidation, eight MAGs contained genes encoding for one of the subunits in either FdoGH or FdhAB while three MAGs (ACT_02, NIT_04, and EUR_14) expressed these genes.

All of the bacterial MAGs and three archaeal MAGs contained and expressed genes involved in carbon fixation (Figure 2, Supplementary Table S3). The ACT_MAGs, CHL_03, NIT_04, and PRO_05 had nearly complete Wood-Ljungdahl pathways (>70%) and all of these MAGs (except for ACT_11) expressed at least one gene involved in the Wood-Ljungdahl pathway. Some MAGs had >50% complete reductive citrate cycle (rTCA) pathways but none of them contained the rTCA autotrophy marker gene coding for ATP-citrate lyase (*aclAB*). Three MAGs (CHL_03, PRO_05, and PRO_16) had a >50% complete dicarboxylate hydroxybutyrate cycle (2HC-4HB) and also had the marker gene *abdfD*. Finally, despite that most MAGs had a >50% complete pathway for the Calvin-Benson-Bassham cycle (CBB), only CRE_07, MIC_10, NAN_12 had the *rubisco* genes. CRE_07 and MIC_10 expressed the *rubisco* genes. It should be noted that Rubisco genes in Archaea typically encode the form III and II/III Rubisco that perform light independent incorporation of CO_2_ into sugars derived from ribonucleotides (Wrighton et al., 2016).

Both bacterial and archaeal MAGs contained and expressed genes involved in fermentation (Figure 2, Supplementary Table S3). Genes involved in transformation of Acetyl-CoA to acetate (reversible) are found in almost all MAGs with the exception of PAT_13 and EUR_06, and seven MAGs expressed gene(s) related to this reaction. Twelve MAGs contained genes to ferment acetate to pyruvate (*porA*), but only ACT_02, NIT_04, EUR_01 and EUR_14 expressed them.

The majority of MAGs contained genes for degrading oligo- and polysaccharides but not all expressed these genes (Supplementary Figure S1). Twelve MAGs expressed at least one gene in a family of glycosyl hydrolases involved in degradation of oligo- and polysaccharides. MAGs ACT_02, CHL_03, NIT_04, PRO_05, EUR_01, and EUR_14 expressed multiple (≥2) gene families. From these MAGs, all except PRO_05 had a relative abundance of ≥3%. The most abundant MAG (EUR_01) had transcripts in gene families GH 15, 57 and 133. GH 15 includes an anomer-inverting glucosidase, trehalase (EC 3.2.1.28 found in *Thermoplasma acidophilium* and *T. volcanium*), and glucoamylase (EC 3.2.1.3 found in *T. acidophilium*). GH57 includes glycosyl hydrolases that act on starch, glycogen, and related oligo- and polysaccharides. Finally, GH133 includes an amylo-alpha-1,6-glucosidase (EC 3.2.1.33).

### Sulfur Cycling

The bacterial MAGs mainly expressed genes involved in dissimilatory sulfate reduction (Figure 3, Supplementary Table S4). The first step in this pathway is the reduction of sulfate to sulfite and catalyzed by *sat* and *aprAB.* The *sat* gene encodes for sulfate adenyltransferase that catalyzes the formation of adenosine 5′-phosphosulfate (APS) from inorganic sulfate and ATP. The *aprAB* gene encodes for an adenosine 5′-phosphosulfate reductase that catalyzes the reduction of APS to sulfite while interacting with the membrane QmoABC complex in the electron transport chain (Anantharaman et al., 2018). Among the eighteen MAGs, thirteen contained *sat* but only four expressed this gene (bacterial MAGs ACT_02, NIT_04, PRO_05, and the archaeal MAG EUR_14). Ten MAGs (including the archaeal CRE_17, CRE_19, and EUR_06) contained genes for AprAB. CRE_19 and all the bacterial MAGs with the exception of CHL_20 expressed *aprAB*. Gene parts of the QmoABC complex were found in almost all bacterial MAGs but in only one archaeal MAG (CRE_19). The genes *qmoABC* were expressed in ACT_02, ACT_11, NIT_04, and PRO_16.

The second step in dissimilatory sulfate reduction is the reduction of sulfite to sulfide (Figure 3, Supplementary Table S4). Genes involved in this step are *dsrAB, dsrC, dsrD, dsrTMK,* and *dsrJOP*. The *dsrAB* genes encode for a dissimilatory sulfite reductase which forms S(II) and S (0) intermediates. DsrAB directly interacts with DsrC for the conversion of the S (0) intermediate to sulfide. The *dsrMKJOP* genes encode for a transmembrane complex involved in electron transport to restore DsrC to its reduced state (Venceslau et al., 2014). DsrMK are the minimum subunits required for electron transfer for sulfite reduction (Pereira et al., 2011). The *dsrD* and *dsrT* genes are likely involved in a regulatory function (Venceslau et al., 2014; Anantharaman et al., 2018). All bacterial MAGs with the exception of CHL_20 contained the genes *dsrAB* but only six MAGs expressed these genes. Among the archaeal MAGs, CRE_19 and EUR_06 contained *dsrAB* but only CRE_19 expressed these genes. Seven MAGs contained *dsrC* but only ACT_02, CHL_03, NIT_04 and CRE_19 expressed this gene. Only bacterial MAGs contained *dsrTMK* and/or *dsrJOP* genes. Finally, *dsrD* was found in all bacterial MAGs with the exception of CHL_20 and PRO_05. The MAGs ACT_02, ACT_11, CHL_03 and NIT_04 expressed *dsrTMK* and *dsrD*.

Other genes involved in reductive S processes are *ttrABC, phsA,* and *hydBG* (Figure 3, Supplementary Table S4)*.* TtrABC are involved in tetrathionate reduction to thiosulfate. PhsA is involved in disproportionation but also in thiosulfate reduction to sulfide. HydBG are involved in sulfur (0) reduction to sulfide (Vigneron et al., 2021). Five MAGs contained and expressed *ttrABC* including the archaeal MAG CRE_19. Three MAGs contained *phsA* and ACT_11 and PRO_05 expressed this gene. Four of the bacterial MAGs contained the *hydBG* genes but only ACT_02 expressed them.

Both bacterial and archaeal MAGs contained genes and/or transcripts involved in sulfide and/or S (0) oxidation (Figure 3, Supplementary Table S4). In this pathway, Sqr oxidizes sulfide to polysulfide, which can be further oxidized to sulfite by the complex DsrABCEFH (Gregersen et al., 2011) or by a periplasmic sulfur dioxygenase (Sdo) (Rohwerder and Sand, 2003). DsrEFH is central in this pathway since it donates sulfur to DsrC (Stockdreher et al., 2012). DsrC then transfers the sulfur to DsrAB acting in the oxidative direction to produce sulfite (Venceslau et al., 2014). On the other hand, sulfur oxidation can also be catalyzed by Sdo, which could also act on perthiols formed by the spontaneous interaction between thiols and elemental sulfur and sulfide (Rohwerder and Sand, 2003). The generated sulfite could be further converted into sulfate by the Sat/AprAB complex (Anantharaman et al., 2018). Nine MAGs had *sqr* and six expressed this gene. Among the ones expressing *sqr* are three of the Euryarchaeota MAGs, including the second most abundant EUR_14. Four MAGs had *sdo* but only the Chloroflexi MAG (CHL_03) expressed this gene. Finally, the Actinobacteria MAGs contained and expressed genes for the complete complex DsrEFH.

A phylogenetic analysis of the *dsrAB* genes found in the MAGs showed that the majority of them clustered with either the bacterial or archaeal reductive type (Supplementary Figure S2). However, the Actinobacteria MAGs each had two copies of the *dsrAB* genes. Copy 1 clustered with the reductive bacterial type while copy 2 clustered with the oxidative bacterial type. From this genomic context analysis, the dsrAB copy 1 from ACT_MAGs were closer to genes coding for DsrMKCBD while copy 2 is closer to the genes coding for DsrEFH*.*

Few MAGs contained genes involved in the oxidation of thiosulfate (Figure 3, Supplementary Table S4). Three bacterial MAGs contained genes for thiosulfate oxidation to tetrathionate (*tsdA* and *doxD*) or sulfate (*soxXAYZB*). The Nitrospirae MAG (NIT_04) is the only MAG that expressed *soxXAYZB*. Rhodanase-like sulfur-transferase (Tst) is another enzyme known for its involvement in thiosulfate oxidation (Vigneron et al., 2021). However, it was also found under sulfur-reducing conditions (Florentino et al., 2019). We found *tst* in three MAGs, two of which (CHL_20 and NIT_04) expressed this gene.

Most of the bacterial MAGs expressed at least one gene involved in dimethyl sulfoxide (DMSO) metabolism (Figure 3, Supplementary Table S4). Ddh specifically catalyzes the transformation of DMSO to dimethyl sulfide (DMS) (Vigneron et al., 2021). Nine MAGs contained genes from the dimethyl sulfoxide dehydrogenase complex (*ddhABC*) and were expressed in five bacterial MAGs (ACT_02, CHL_03, CHL_20, NIT_04 and PRO_05). Seven MAGs contained *dmoB,* which encodes for a dimethyl-sulfide monooxygenase subunit B, but only NIT_04 expressed this gene. Only one MAG (CHL_03) contained *dmsAB,* encoding for an anaerobic dimethyl sulfoxide reductase, but these genes were not expressed.

### Other Respiratory Pathways

Bacterial MAGs contained genes involved in oxidative phosphorylation, oxygen reduction, hydrogen metabolism, nitrate/nitrite reduction, and Fe(III) reduction (Figure 4, Supplementary Table S5). All MAGs except for PAT_13 contained genes involved in the complex I (*nuoABC*) and complex V (*atpAD* F-type) for oxidative phosphorylation, and ACT_02, CHL_03, and NIT_04 expressed these genes. Most bacterial MAGs contained a gene involved in oxygen reduction (*cydAB, cyoABCD, ccoNOP,* and *coxAB*). ACT_02 expressed *ccoNOP* and NIT_04 expressed *cydAB*. Five of the bacterial MAGs contained genes involved in hydrogen metabolism (*nife-group1*). NIT_04 and PRO_05 expressed *nife-group-1* involved in hydrogen oxidation. Three bacterial MAGs contained genes involved in nitrate reduction (*narGH*) and NIT_04 expressed *narGH*. The gene *nrfA* involved in nitrite reduction to ammonia was expressed by PRO_16. Finally, the gene *mtrC* involved in Fe(III) reduction was expressed by NIT_04.

In contrast to the bacterial MAGs, archaeal MAGs only contained genes involved in oxidative phosphorylation (Figure 4, Supplementary Table S5). The Euryarchaeota-MAGs, EUR_01, EUR_06, and EUR_14 contained *nuoABC* genes. All archaeal MAGs except for NAN_12 contained *atpAB* V/A-type genes, and these were expressed by CRE_19, EUR_01, and EUR_14. These three MAGs and EUR_15 also expressed a gene from the CydAB complex, a high affinity oxygen reductase associated with oxygen-limited conditions (Borisov et al., 2011).

## Discussion

It is worthwhile to note how the current study relates to previous work done using these same data. In Ayala-Muñoz et al. (Ayala-Muñoz et al., 2020), we used whole community metagenomic and metatranscriptomic data to examine the potential and expression of known metal resistance genes (MRGs) in all three layers of CM. We compared metal resistance across communities using a curated list of protein-coding MRGs with KEGG Orthology identifiers (KOs) and found broad differences in the metal resistance strategies expressed by Eukaryotes, Bacteria, and Archaea. In Ayala-Muñoz et al. (Ayala-Muñoz et al., 2022), we used a combination of amplicon sequencing, metagenomics and metatranscriptomics to perform a taxonomically-resolved analysis of microbial contributions to carbon, sulfur, iron, and nitrogen cycling in all three layers of CM. We calculated thermodynamic potentials for various metabolisms based on geochemical conditions in each layer and compared these data to corresponding gene/pathway expressions to construct a functional biogeochemical model of the lake. In this current study, we focus solely on the deep layer of CM and the potential and expression of MAGs recovered from this layer.

It is also important to note that our analysis of gene expression is limited by the fact that we are reporting results from only one metatranscriptome. Furthermore, our analysis is also limited by the completeness of any one MAG. If the expression of a gene was detected, we can conclude with certainty that it was expressed, however, the absence of expression could come from a detection issue or biological heterogeneity that we were unable to account for. Given these premises, our discussion focuses largely on pathways that were present and expressed. We highlight lacking genes and associated transcripts only if particularly remarkable findings could serve as starting point for future research.

### Novel Acidophilic Bacteria Contributing to Biosulfidogenesis

Microbial strains unrelated to cultured representatives of known sulfate reducing bacteria actively contributed to biosulfidogenesis in the deep layer of CM and were metabolically versatile. Six novel strains represented by bacterial MAGs ACI_09, ACT_02, ACT_11, CHL_03, NIT_04, and PRO_16 contained genes for the reduction of sulfate to sulfide, including the *dsrD* gene (Figure 3). Populations represented by these six MAGs accounted for roughly 15% of the microbial community (Figure 1). The *dsrD* gene is an important marker for sulfate reduction given its absence in the genomes of sulfide oxidizing organisms (Rabus et al., 2015; Anantharaman et al., 2018). Reduction of sulfate could be coupled to oxidation of organic carbon or to hydrogen and indeed, these six MAGs expressed genes for acetate (Figure 2) or H_2_ oxidation (Figure 4). All of these MAGs except for ACI_09 contained a nearly complete Wood-Ljungdhal pathway, suggesting that they could fix inorganic carbon when organic carbon (e.g., organic acids) is not available (Figure 2). This hypothesis is supported by the high concentration of CO_2_ (29 mM) available for the microbial populations in the deep layer (Sánchez-España et al., 2020b). Some of these MAGs also contained genes involved in sulfide, S (0), or thiosulfate oxidation (Figure 3), which could be coupled with oxygen, nitrate, or ferric iron reduction (Figure 4). Our results suggest that metabolic versatility is beneficial to these populations, and along with evidence for sulfide oxidation in the abundant *Thermoplasmatales* populations, points toward transient changes in redox potential and dissolved oxygen concentrations in the CM deep layer.

**FIGURE 5:**
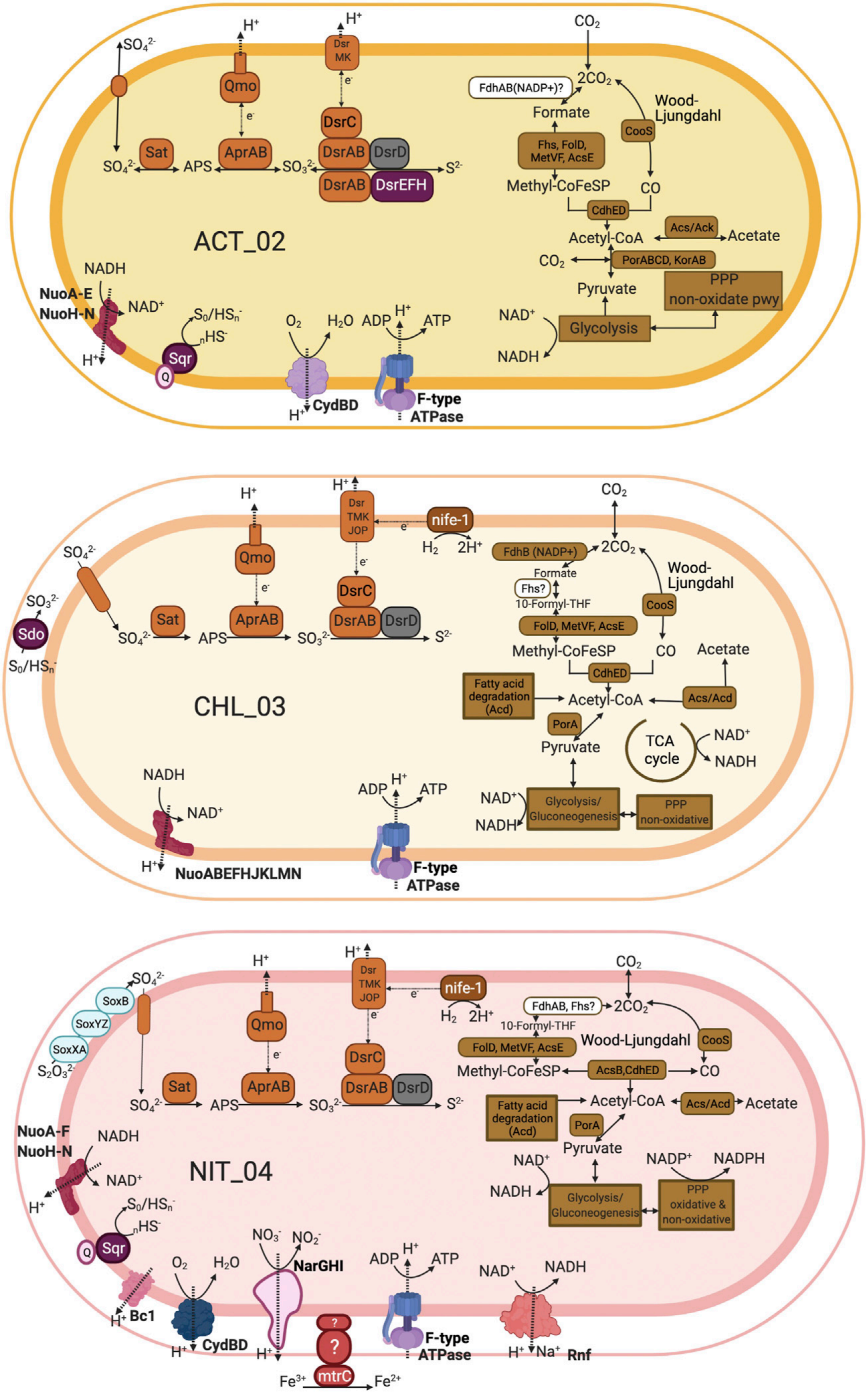
Conceptual metabolic representations of the three MAGs most likely contributing to *in situ* activity for biosulfidogenesis in the CM deep layer. ACT_02 is most taxonomically related to an uncultured *Actinobacteria* from the class *Thermoleophilia* and is capable of sulfate reduction (reductive-type *dsrAB*) and sulfide oxidation (oxidative-type *dsrAB*). CHL_03 is most closely related to an uncultured *Chloroflexi* from the class *Dehalococcoidia*. It represents a population of fermenters capable of sulfate reduction. NIT_04 is most closely related to an uncultured *Nitrospirae* from the class *Thermodesulfovibrionia*. It is capable of sulfate reduction but also sulfide and thiosulfate oxidation potentially coupled with oxygen, nitrate and Fe(III) reduction. Created with Biorender.com.

Remarkably, the most abundant bacterial MAGs (ACT_02, CHL_03, and NIT_04) expressed genes for almost the entire set of enzymes related to biosulfidogenesis (Figure 3), and represented novel taxa. The taxonomic analysis of these MAGs with GTDB-Tk did not report ANI ≥96.5% to any genome found in the Genome Taxonomy Database. ACT_02 (*Actinobacteria,* class *Thermoleophilia* (Figure 1, Supplementary Table S1)) populations made up roughly 3% of the total community. The closest genome related to this MAG was *Adlercreutzia equolifaciens DSM 19450* with an AAI (average aminoacid identity) of 43% according to the NCBI_Prok database in the Microbial Genome Atlas (Supplementary Table S2). The few acidophilic *Actinobacteria* described to date include ferrous-iron oxidizers (e.g., *Acidimicrobium* ferrooxidans), ferric-iron reducers (*Aciditerrimonas ferrireducens*), and/or sulfur-oxidizers (*Acidithiomicrobium* spp.) (Clark and Norris, 1996; Davis-Belmar and Norris, 2009; Itoh et al., 2011), and none with the ability for performing biosulfidogenesis, only, the neutrophile *Gordonibacter pamelaeae* is among the few cultivated *Actinobacteria* with *dsrAB* genes (Müller et al., 2015) along with other neutrophilic, non-cultured *Actinobacteria* (Anantharaman et al., 2018)*.* Within the *Thermoleophilia* class, the few known representatives, retrieved from either hot springs or soil samples, have no reported contribution to sulfur cycling (Hu et al., 2019). However, the metagenomic evidence found in this work suggests that ACT_02 populations are organoheterotrophs able to reduce sulfate and oxidize sulfide (Figure 5). They have the potential to oxidize pyruvate to acetyl-CoA by a pyruvate ferredoxin oxidoreductase (PorABCD) and a 2-oxoglutarate/2-oxoacid ferredoxin oxidoreductase (KorAB). ACT_02 populations also have an *acs* involved in Acetyl_CoA generation from acetate, complete glycolysis (Embden-Meyerhof) and non-oxidative pentose phosphate pathways, and all the genes encoding for the methyl and carbonyl branches of the Wood-Ljungdhal pathway except for *fdhAB.* The lack of the genes encoding for Pta and AcdA suggests that ACT_02 could not grow autotrophically (Schuchmann and Müller, 2014). Energy generation is supported by the NADH quinone dehydrogenase complex (Nuo) and dissimilatory sulfate reduction (Sat, AprAB, Qmo, DsrABCDMK). ACT_02 populations were also capable of sulfide oxidation (Sqr and DsrEFH*).* They have a second (oxidative) copy of *dsrAB* located closer to the genes *dsrEFH* (Supplementary Figure S2), and the genes necessary for O_2_ respiration, including CydBD.

CHL_03 (*Chloroflexi,* class *Dehalococcoidia* (Figure 1, Supplementary Table S1)) populations made up roughly 3% of the total community. The closest genome related to this MAG was *Dehalococcoides mccartyi* VS*. NC 013552* with an AAI of 44% according to the NCBI_Prok database in the Microbial Genome Atlas (Supplementary Table S2). Previous studies presented evidence of acidophilic *Chloroflexi* populations in environments including acidic pit lakes (Santofimia et al., 2013; García-Moyano et al., 2015; Mesa et al., 2017), but their metabolisms has not determined. A single-cell genome from this class was retrieved from a marine subsurface sediment and is the only reported genome in the class to date with *dsrAB* genes (Wasmund et al., 2016). Analysis of the CHL_03 MAG points to a fermentative lifestyle with the additional ability to reduce sulfate (Figure 5). CHL_03 lacked four out of ten subunits of the NADH-ubiquinone oxidoreductase (Nuo) complex. The interaction of NuoEF with its membrane anchor NuoLMN subunits is improbable given the lack of the connecting subunits NuoCDGI. CHL_03 also lacked standard electron transport chain components including cytochrome c reductases (Complex III) and cytochrome c oxidases (Complex IV). It contained *acs* and *porA* involved in acetate and pyruvate oxidation to Acetyl-CoA. CHL_03 had also an incomplete TCA cycle with a lack of citrate synthase, citrate lyase, and aconitate hydratase. CHL_03 contained a complete set of genes involved in glycolysis and gluconeogenesis. Such evidence suggests a primarily fermentative metabolism relying on sugars for energy generation. Acetyl-CoA could also be generated by fatty acid degradation and by CO_2_ reduction via the Wood-Ljungdahl pathway. Autotrophic growth by acetogenesis is potentially feasible thanks to ATP generated by AcdA*.* CHL_03 populations could also carry out sulfate reduction (Sat, AprAB, Qmo, DsrABCDTMKJOP) coupled to hydrogen oxidation (nife-1) to generate a proton motive force which fuels ATP formation by an F-type ATPase. CHL_03 MAG was 97.7% complete and, although unlikely, it is possible that the lacking genes were missed due to the incompleteness of the genome.

NIT_04 (*Nitrospirae*, Figure 1, Supplementary Table S1) populations made up roughly 3% of the total community. The closest genome related to this MAG was *Thermodesulfovibrio yellowstonii DSM_11347_NC_011296* with an AAI of 45.97% according to the NCBI_Prok database in the Microbial Genome Atlas (Supplementary Table S2). The most renowned acidophilic isolates from this phylum are *Leptospirillum* strains known for their low pH iron-oxidation capability (Méndez-García et al., 2015). However, NIT_04 populations were affiliated with the genus *Thermodesulfovibrio.* To date all *Thermodesulfovibrio* isolates are neutrophiles or alkaliphiles (Henry et al., 1994; Frank et al., 2016), there are no reported genomes of acidophilic *Thermodesulfovibrio* strains. However there is molecular evidence of acidophilic *Thermodesulfovibrio* presence in at least one acidic environment from anoxic hot spring sediments (Willis et al., 2019). Some members of this genus such as *Thermodesulfovibrio thiophilus* (Sekiguchi et al., 2008) have the reductive bacterial-type DsrAB (Müller et al., 2015). NIT_04 populations are organoheterotrophs that can reduce sulfate and oxidize sulfide and thiosulfate (Figure 5). Acetate and pyruvate could be oxidized to acetyl-CoA thanks to the presence of Acs and PorA. Fatty acid degradation could also be used to produce Acetyl-CoA. NIT_04 also contained complete sets of genes for glycolysis, gluconeogenesis, and the pentose phosphate pathway (oxidative and non-oxidative branches). NIT_04 had an incomplete methyl branch and a complete carbonyl branch of the Wood-Ljungdahl pathway. However, NIT_04 also had genes encoding for MtaA, a methyl transferase involved in the methyl branch of the WL (not shown in Figure 5), and AcdA involved in energy generation, suggesting that NIT_04 could utilize methyl compounds to get energy using the Wood-Ljungdahl pathway. NIT_04 reduced sulfate all the way to sulfide (Sat, AprAB, Qmo, DsrABCDTMKJOP) coupled to hydrogen oxidation (nife-group-1). NIT_04 also expressed *sqr,* involved in sulfide oxidation, and the Sox complex (*soxXAYZB*), involved in thiosulfate oxidation. Sulfide or thiosulfate oxidation could be coupled to oxygen respiration (CydBD). Coupling of electron transfer to energy conservation could be mediated in NIT_04 by an H/Na-pumping Rnf complex, an NADH-quinone oxidoreductase (Nuo) and an F-type ATPase synthase. Genes involved in nitrate reduction (*narGH*) and metal reduction (*mtrC*) were also found, providing evidence of the potential to use a diverse range of electron acceptors in respiration.

### Biotechnological Implications: *In situ* Stimulation of Biosulfidogenesis

Cueva de la Mora (CM) is one the world’s most studied AMD sites and represents a model system for discovering novel acidophilic and acid-tolerant organisms. While restoration of water quality to pre-mining conditions may be financially prohibitive, stimulation of biosulfidogenesis in the deep layer of permanently stratified pit lakes such as CM may be a cost-effective means to limit environmental risks posed by elevated metal (loid) concentrations. The most abundant populations in the deep layer are *Thermoplamatales*-group archaea that likely scavenge organic carbon and potentially oxidize sulfide using trace concentrations of oxygen. Therefore, we focus our discussion on MAGs representing abundant populations that contribute to biosulfidogenesis under *in situ* conditions. Based on our findings, we speculate on how biosulfidogenesis could be enhanced in meromictic acidic pit lakes.

Our results strongly suggest that organic carbon (C_org_) additions will be required to stimulate biosulfidogenesis in the CM deep layer, both to fuel sulfide production directly and to consume oxidants (e.g., O_2_) which, although present at low concentrations, currently allow for the rapid re-oxidation of reduced sulfur compounds. A stumbling block in historical biosulfidogenesis efforts in AMD systems is the high toxicity of organic acids at low pH (Pinhal et al., 2019). Depending on metabolic networks in the C_org_-amended community, this toxicity may become limiting for sulfide production by organic acid-oxidizing sulfate-reducing microbial populations. In this case, the addition of S (0) in combination with C_org_ at lower loadings (to fuel S (0) reduction) or no C_org_ addition (to fuel S (0) disproportionation) are alternative strategies that may result in higher sulfide production due to lower organic acid toxicity. These alternative strategies will be tested in future laboratory work with enrichments from the CM deep layer.

## Conclusion

We conducted a population-resolved omics analysis of the deep layer of the acidic pit lake Cueva de la Mora. We found that populations represented by three MAGs belonging to the *Thermoplasmatales* archaea dominate the microbial community. A careful analysis of the functional potential of the *Thermoplasmatales* MAGs revealed that these organisms are likely carbon scavengers and capable of oxidizing sulfide to S (0) coupled with the reduction of O_2._ Oxygen levels in the deep layer are perennially below detection (<0.02 mg/L O_2_), thus these populations either perform sulfide oxidation under very low oxygen concentrations, or their activity and growth is triggered by episodic oxygen additions to the deep layer by groundwater injection. The S cycle in Cueva de la Mora deep water is completed by the presence of abundant bacteria capable of biosulfidogenesis belonging to novel taxa from the *Actinobacteria, Chloroflexi*, and *Nitrospirae* phyla*.*

Biosulfidogenesis represents an avenue for acid pit lake remediation as sulfide can react with dissolved heavy metal (loid)s to form sparingly soluble metal sulfides. In this case, remediation amounts to sequestration of metal (loid)s in lake sediments. Removal of metal (loid)s from the lake water would reduce off-site contaminant transport in polluted groundwater and springs and associated risks to humans and ecosystems. Based on the analysis of MAGs recovered from the deep layer of CM, biosulfidogenesis could be stimulated in several ways. Because trace levels of oxygen may be present in the deep layer, the addition of organic carbon would help to consume trace oxygen and limit sulfide oxidation. However, organic carbon additions could produce organic acids that are toxic at low pH. An alternative strategy is to add S (0) to promote S (0) disproportionation either in place of or in combination with organic carbon. Future work with whole communities collected from the deep layer of CM will be used to evaluate these sulfide-producing community processes.

## Data Availability

Raw data for metagenomes and metatranscriptomes are available in the SRA database as bioproject PRJNA646106. This bioproject also includes the MAGs in fasta files.

## References

[B1] Alazard D. Joseph M. Battaglia-Brunet F. Cayol J.-L. Ollivier B. (2010). Desulfosporosinus Acidiphilus Sp. nov.: a Moderately Acidophilic Sulfate-Reducing Bacterium Isolated from Acid Mining Drainage Sediments. Extremophiles 14 (3), 305–312. 10.1007/s00792-010-0309-4 20358236

[B2] Anantharaman K. Hausmann B. Jungbluth S. P. Kantor R. S. Lavy A. Warren L. A. (2018). Expanded Diversity of Microbial Groups that Shape the Dissimilatory Sulfur Cycle. Isme J. 12 (7), 1715–1728. 10.1038/s41396-018-0078-0 29467397 PMC6018805

[B3] Aramaki T. Blanc-Mathieu R. Endo H. Ohkubo K. Kanehisa M. Goto S. (2019). KofamKOALA: KEGG Ortholog Assignment Based on Profile HMM and Adaptive Score Threshold. bioRxiv, 36, 602110. 10.1093/bioinformatics/btz859 PMC7141845 31742321

[B4] Ayala-Muñoz D. Burgos W. D. Sánchez-España J. Couradeau E. Falagán C. Macalady J. L. (2020). Metagenomic and Metatranscriptomic Study of Microbial Metal Resistance in an Acidic Pit Lake. Microorganisms 8 (9), 1350. 10.3390/microorganisms8091350 PMC7563247 32899650

[B5] Ayala-Muñoz D. Macalady J. L. Sánchez-España J. Falagán C. Couradeau E. Burgos W. D. (2022). Omics-enabled Analysis of Biogeochemical Cycling in the Water Column of a Meromictic Acidic Pit Lake. ISME J. in revision April 2022.

[B6] Borisov V. B. Gennis R. B. Hemp J. Verkhovsky M. I. (2011). The Cytochrome Bd Respiratory Oxygen Reductases. Biochimica Biophysica Acta (BBA) - Bioenergetics 1807 (11), 1398–1413. 10.1016/j.bbabio.2011.06.016 PMC3171616 21756872

[B7] Chaumeil P-A. Mussig A. J. Hugenholtz P. Parks D. H. (2019). GTDB-tk: a Toolkit to Classify Genomes with the Genome Taxonomy Database. Bioinformatics 36, 1925–1927. 10.1093/bioinformatics/btz848 PMC7703759 31730192

[B8] Clark D. A. Norris P. R. (1996). Acidimicrobium Ferrooxidans Gen. nov., Sp. nov.: Mixed-Culture Ferrous Iron Oxidation with Sulfobacillus Species. Microbiology 142 (4), 785–790. 10.1099/00221287-142-4-785 33725781

[B9] Davis-Belmar C. S. Norris P. R. (2009). Ferrous Iron and Pyrite Oxidation by "Acidithiomicrobium" Species. Amr 71-73, 271–274. 10.4028/www.scientific.net/amr.71-73.271

[B10] Eddy S. R. (2011). Accelerated Profile HMM Searches. PLoS Comput. Biol. 7 (10), e1002195. 10.1371/journal.pcbi.1002195 22039361 PMC3197634

[B11] Falagán C. Sánchez‐España J. Johnson D. B. (2014). New Insights into the Biogeochemistry of Extremely Acidic Environments Revealed by a Combined Cultivation‐based and Culture‐independent Study of Two Stratified Pit Lakes. FEMS Microbiol. Ecol. 87 (1), 231–243. 10.1111/1574-6941.12218 24102574

[B12] Finn R. D. Bateman A. Clements J. Coggill P. Eberhardt R. Y. Eddy S. R. (2014). Pfam: the Protein Families Database. Nucleic Acids Res. 42, D222–D230. 10.1093/nar/gkt1223 24288371 PMC3965110

[B13] Florentino A. P. Stams A. J. Sánchez-Andrea I. (2017). Genome Sequence of Desulfurella Amilsii Strain TR1 and Comparative Genomics of Desulfurellaceae Family. Front. Microbiol. 8, 222. 10.3389/fmicb.2017.00222 28265263 PMC5317093

[B14] Florentino A. P. Pereira I. A. C. Boeren S. van den Born M. Stams A. J. M. Sánchez-Andrea I. (2019). Insight into the Sulfur Metabolism ofDesulfurella Amilsiiby Differential Proteomics. Environ. Microbiol. 21 (1), 209–225. 10.1111/1462-2920.14442 30307104 PMC6378623

[B15] Frank Y. A. Kadnikov V. V. Lukina A. P. Banks D. Beletsky A. V. Mardanov A. V. (2016). Characterization and Genome Analysis of the First Facultatively Alkaliphilic Thermodesulfovibrio Isolated from the Deep Terrestrial Subsurface. Front. Microbiol. 7, 2000. 10.3389/fmicb.2016.02000 28066337 PMC5165239

[B16] Frolov E. N. Kublanov I. V. Toshchakov S. V. Samarov N. I. Novikov A. A. Lebedinsky A. V. (2017). Thermodesulfobium Acidiphilum Sp. nov., a Thermoacidophilic, Sulfate-Reducing, Chemoautotrophic Bacterium from a Thermal Site. Int. J. Syst. Evol. Microbiol. 67 (5), 1482–1485. 10.1099/ijsem.0.001745 27995866

[B17] García-Moyano A. Austnes A. E. Lanzén A. González-Toril E. Aguilera Á. Øvreås L. (2015). Novel and Unexpected Microbial Diversity in Acid Mine Drainage in Svalbard (78° N), Revealed by Culture-independent Approaches. Microorganisms 3 (4), 667–694. 10.3390/microorganisms3040667 27682111 PMC5023264

[B18] Gregersen L. H. Bryant D. A. Frigaard N. U. (2011). Mechanisms and Evolution of Oxidative Sulfur Metabolism in Green Sulfur Bacteria. Front. Microbiol. 2, 116. 10.3389/fmicb.2011.00116 21833341 PMC3153061

[B19] Haft D. H. Selengut J. D. White O. (2003). The TIGRFAMs Database of Protein Families. Nucleic acids Res. 31 (1), 371–373. 10.1093/nar/gkg128 12520025 PMC165575

[B20] Henry E. A. Devereux R. Maki J. S. Gilmour C. C. Woese C. R. Mandelco L. (1994). Characterization of a New Thermophilic Sulfate-Reducing Bacterium. Arch. Microbiol. 161 (1), 62–69. 10.1007/bf00248894 11541228

[B21] Hu D. Zang Y. Mao Y. Gao B. (2019). Identification of Molecular Markers that Are Specific to the Class Thermoleophilia. Front. Microbiol. 10, 1185. 10.3389/fmicb.2019.01185 31178855 PMC6544083

[B22] Hyatt D. Chen G.-L. LoCascio P. F. Land M. L. Larimer F. W. Hauser L. J. (2010). Prodigal: Prokaryotic Gene Recognition and Translation Initiation Site Identification. BMC Bioinforma. 11, 119. 10.1186/1471-2105-11-119 PMC2848648 20211023

[B23] Itoh T. Yamanoi K. Kudo T. Ohkuma M. Takashina T. (2011). Aciditerrimonas Ferrireducens Gen. nov., Sp. nov., an Iron-Reducing Thermoacidophilic Actinobacterium Isolated from a Solfataric Field. Int. J. Syst. Evol. Microbiol. 61 (6), 1281–1285. 10.1099/ijs.0.023044-0 20639230

[B24] Jain C. Rodriguez-R L. M. Phillippy A. M. Konstantinidis K. T. Aluru S. (2018). High Throughput ANI Analysis of 90K Prokaryotic Genomes Reveals Clear Species Boundaries. Nat. Commun. 9 (1), 5114. 10.1038/s41467-018-07641-9 30504855 PMC6269478

[B25] JGI. (2017). BBmap: Joint Genome Institute JGI; [BBmap]. JGI. Available from: https://jgi.doe.gov/data-and-tools/bbtools/bb-tools-user-guide/bbmap-guide/ .(Accessed.September.6.2017).

[B26] Johnson D. B. Sánchez-Andrea I. (2019). “Dissimilatory Reduction of Sulfate and Zero-Valent Sulfur at Low pH and its Significance for Bioremediation and Metal Recovery”. Advances in Microbial Physiology. 75, 205–231. 10.1016/bs.ampbs.2019.07.002 31655738

[B27] Johnson D. B. Santos A. L. (2020). “Biological Removal of Sulfurous Compounds and Metals from Inorganic Wastewaters,” in Environmental Technologies to Treat Sulfur Pollution: Principles and Engineering. Editor Lens P. N. L. (London: IWA Publishing), 0. 10.2166/9781789060966_0215

[B28] Kanehisa M. Sato Y. (2020). KEGG Mapper for Inferring Cellular Functions from Protein Sequences. Protein Sci. 29 (1), 28–35. 10.1002/pro.3711 31423653 PMC6933857

[B29] Kang D. D. Li F. Kirton E. Thomas A. Egan R. An H. (2019). MetaBAT 2: an Adaptive Binning Algorithm for Robust and Efficient Genome Reconstruction from Metagenome Assemblies. PeerJ 7, e7359. 10.7717/peerj.7359 31388474 PMC6662567

[B30] Kopylova E. Noé L. Touzet H. (2012). SortMeRNA: Fast and Accurate Filtering of Ribosomal RNAs in Metatranscriptomic Data. Bioinformatics 28 (24), 3211–3217. 10.1093/bioinformatics/bts611 23071270

[B31] Kurosawa N. Itoh Y. H. Iwai T. Sugai A. Uda I. Kimura N. (1998). Sulfurisphaera Ohwakuensis Gen. nov., Sp. nov., a Novel Extremely Thermophilic Acidophile of the Order Sulfolobales. Int. J. Syst. Bacteriol. 48 Pt 2, 451–456. 10.1099/00207713-48-2-451 9731283

[B32] Langmead B. Trapnell C. Pop M. Salzberg S. L. (2009). Ultrafast and Memory-Efficient Alignment of Short DNA Sequences to the Human Genome. Genome Biol. 10 (3), R25. 10.1186/gb-2009-10-3-r25 19261174 PMC2690996

[B33] Letunic I. Bork P. (2016). Interactive Tree of Life (iTOL) V3: an Online Tool for the Display and Annotation of Phylogenetic and Other Trees. Nucleic Acids Res. 44 (W1), W242–W245. 10.1093/nar/gkw290 27095192 PMC4987883

[B34] Li D. Luo R. Liu C.-M. Leung C.-M. Ting H.-F. Sadakane K. (2016). MEGAHIT v1.0: A Fast and Scalable Metagenome Assembler Driven by Advanced Methodologies and Community Practices. Methods 102, 3–11. 10.1016/j.ymeth.2016.02.020 27012178

[B35] Lombard V. Golaconda Ramulu H. Drula E. Coutinho P. M. Henrissat B. (2013). The Carbohydrate-Active Enzymes Database (CAZy) in 2013. Nucl. Acids Res. 42 (D1), D490–D495. 10.1093/nar/gkt1178 24270786 PMC3965031

[B36] Mardanov A. V. Panova I. A. Beletsky A. V. Avakyan M. R. Kadnikov V. V. Antsiferov D. V. (2016). Genomic Insights into a New Acidophilic, Copper-Resistant Desulfosporosinus Isolate from the Oxidized Tailings Area of an Abandoned Gold Mine. FEMS Microbiol. Ecol. 92 (8), fiw111. 10.1093/femsec/fiw111 27222219

[B37] Méndez-García C. Peláez A. I. Mesa V. Sánchez J. Golyshina O. V. Ferrer M. (2015). Microbial Diversity and Metabolic Networks in Acid Mine Drainage Habitats. Front. Microbiol. 6, 475. 10.3389/fmicb.2015.00475 26074887 PMC4448039

[B38] Mesa V. Gallego J. L. R. González-Gil R. Lauga B. Sánchez J. Méndez-García C. (2017). Bacterial, Archaeal, and Eukaryotic Diversity across Distinct Microhabitats in an Acid Mine Drainage. Front. Microbiol. 8, 1756. 10.3389/fmicb.2017.01756 28955322 PMC5600952

[B39] Miller C. S. Baker B. J. Thomas B. C. Singer S. W. Banfield J. F. (2011). EMIRGE: Reconstruction of Full-Length Ribosomal Genes from Microbial Community Short Read Sequencing Data. Genome Biol. 12 (5), R44. 10.1186/gb-2011-12-5-r44 21595876 PMC3219967

[B40] Mincer T. J. Aicher A. C. (2016). Methanol Production by a Broad Phylogenetic Array of Marine Phytoplankton. PLOS ONE 11 (3), e0150820. 10.1371/journal.pone.0150820 26963515 PMC4786210

[B41] Mori K. Kim H. Kakegawa T. Hanada S. (2003). A Novel Lineage of Sulfate-Reducing Microorganisms: Thermodesulfobiaceae Fam. nov., Thermodesulfobium Narugense , Gen. nov., Sp. nov., a New Thermophilic Isolate from a Hot Spring. Extremophiles 7 (4), 283–290. 10.1007/s00792-003-0320-0 12910388

[B42] Müller A. L. Kjeldsen K. U. Rattei T. Pester M. Loy A. (2015). Phylogenetic and Environmental Diversity of DsrAB-type Dissimilatory (Bi)sulfite Reductases. ISME J. 9 (5), 1152–1165. 10.1038/ismej.2014.208 25343514 PMC4351914

[B43] Ňancucheo I. Johnson D. B. (2012). Selective Removal of Transition Metals from Acidic Mine Waters by Novel Consortia of Acidophilic Sulfidogenic Bacteria. Microb. Biotechnol. 5 (1), 34–44. 10.1111/j.1751-7915.2011.00285.x 21895996 PMC3815270

[B44] Nurk S. Meleshko D. Korobeynikov A. Pevzner P. A. (2017). metaSPAdes: a New Versatile Metagenomic Assembler. Genome Res. 27 (5), 824–834. 10.1101/gr.213959.116 28298430 PMC5411777

[B45] Olm M. R. Brown C. T. Brooks B. Banfield J. F. (2017). dRep: a Tool for Fast and Accurate Genomic Comparisons that Enables Improved Genome Recovery from Metagenomes through De-replication. Isme J. 11 (12), 2864–2868. 10.1038/ismej.2017.126 28742071 PMC5702732

[B46] Panova I. A. Ikkert O. Avakyan M. R. Kopitsyn D. S. Mardanov A. V. Pimenov N. V. (2021). Desulfosporosinus Metallidurans Sp. nov., an Acidophilic, Metal-Resistant Sulfate-Reducing Bacterium from Acid Mine Drainage. Int. J. Syst. Evol. Microbiol. 71 (7). 1. 10.1099/ijsem.0.004876 34255623

[B47] Pereira I. A. Ramos A. R. Grein F. Marques M. C. Da Silva S. M. Venceslau S. S. (2011). A Comparative Genomic Analysis of Energy Metabolism in Sulfate Reducing Bacteria and Archaea. Front. Microbiol. 2, 69. 10.3389/fmicb.2011.00069 21747791 PMC3119410

[B48] Quast C. Pruesse E. Yilmaz P. Gerken J. Schweer T. Yarza P. (2013). The SILVA Ribosomal RNA Gene Database Project: Improved Data Processing and Web-Based Tools. Nucleic Acids Res. 41 (D1), D590–D596. 10.1093/nar/gks1219 23193283 PMC3531112

[B49] Rabus R. Venceslau S. S. Wöhlbrand L. Voordouw G. Wall J. D. Pereira I. A. C. (2015). “A Post-Genomic View of the Ecophysiology, Catabolism and Biotechnological Relevance of Sulphate-Reducing Prokaryotes. Adv. Microb. Physiology 66, 55–321. 10.1016/bs.ampbs.2015.05.002 26210106

[B50] Rodriguez-R L. M. Gunturu S. Harvey W. T. Rosselló-Mora R. Tiedje J. M. Cole J. R. (2018). The Microbial Genomes Atlas (MiGA) Webserver: Taxonomic and Gene Diversity Analysis of Archaea and Bacteria at the Whole Genome Level. Nucleic acids Res. 46 (W1), W282–W288. 10.1093/nar/gky467 29905870 PMC6031002

[B51] Rohwerder T. Sand W. (2003). The Sulfane Sulfur of Persulfides Is the Actual Substrate of the Sulfur-Oxidizing Enzymes from Acidithiobacillus and Acidiphilium Spp. Microbiol. Read. 149, 1699–1710. 10.1099/mic.0.26212-0 12855721

[B52] Salazar G. Paoli L. Alberti A. Huerta-Cepas J. Ruscheweyh H.-J. Cuenca M. (2019). Gene Expression Changes and Community Turnover Differentially Shape the Global Ocean Metatranscriptome. Cell. 179 (5), 1068–1083. e21. 10.1016/j.cell.2019.10.014 31730850 PMC6912165

[B53] Sánchez-Andrea I. Stams A. J. M. Hedrich S. Ňancucheo I. Johnson D. B. (2015). Desulfosporosinus Acididurans Sp. nov.: an Acidophilic Sulfate-Reducing Bacterium Isolated from Acidic Sediments. Extremophiles 19 (1), 39–47. 10.1007/s00792-014-0701-6 25370366

[B54] Sánchez-España J. Pamo E. L. Diez M. Santofimia E. (2009). Physico-chemical gradients and meromictic stratification in Cueva de la Mora and other acidic pit lakes of the Iberian Pyrite Belt. Mine Water Environ. 28 (1), 15–29. 10.1007/s10230-008-0059-z

[B55] Sánchez-España J. Yusta I. Ilin A. van der Graaf C. Sánchez-Andrea I. (2020). Microbial Geochemistry of the Acidic Saline Pit Lake of Brunita Mine (La Unión, SE Spain). Spain: Mine Water and the Environment, 1–21.

[B56] Sánchez-España J. Falagán C. Ayala D. Wendt-Potthoff K. (2020). Adaptation of Coccomyxa Sp. To Extremely Low Light Conditions Causes Deep Chlorophyll and Oxygen Maxima in Acidic Pit Lakes. Microorganisms 8 (8), 1218. 10.3390/microorganisms8081218 PMC7465793 32796657

[B57] Santofimia E. González-Toril E. López-Pamo E. Gomariz M. Amils R. Aguilera Á. (2013). Microbial Diversity and its Relationship to Physicochemical Characteristics of the Water in Two Extreme Acidic Pit Lakes from the Iberian Pyrite Belt (SW Spain). Plos One 8 (6), e66746. 10.1371/journal.pone.0066746 23840525 PMC3694112

[B58] Schuchmann K. Müller V. (2014). Autotrophy at the Thermodynamic Limit of Life: a Model for Energy Conservation in Acetogenic Bacteria. Nat. Rev. Microbiol. 12 (12), 809–821. 10.1038/nrmicro3365 25383604

[B59] Segerer A. Neuner A. Kristjansson J. K. Stetter K. O. (1986). Acidianus Infernus Gen. nov., Sp. nov., and Acidianus Brierleyi Comb. nov.: Facultatively Aerobic, Extremely Acidophilic Thermophilic Sulfur-Metabolizing Archaebacteria. Int. J. Syst. Bacteriol. 36 (4), 559–564. 10.1099/00207713-36-4-559

[B60] Segerer A. Langworthy T. A. Stetter K. O. (1988). Thermoplasma Acidophilum and Thermoplasma Volcanium Sp. Nov. From Solfatara Fields. Syst. Appl. Microbiol. 10 (2), 161–171. 10.1016/s0723-2020(88)80031-6

[B61] Segerer A. H. Trincone A. Gahrtz M. Stetter K. O. (1991). Stygiolobus Azoricus Gen. nov., Sp. Nov. Represents a Novel Genus of Anaerobic, Extremely Thermoacidophilic Archaebacteria of the Order Sulfolobales. Int. J. Syst. Bacteriol. 41 (4), 495–501. 10.1099/00207713-41-4-495

[B62] Sekiguchi Y. Muramatsu M. Imachi H. Narihiro T. Ohashi A. Harada H. (2008). Thermodesulfovibrio Aggregans Sp. Nov. And Thermodesulfovibrio Thiophilus Sp. nov., Anaerobic, Thermophilic, Sulfate-Reducing Bacteria Isolated from Thermophilic Methanogenic Sludge, and Emended Description of the Genus Thermodesulfovibrio. Int. J. Syst. Evol. Microbiol. 58 (11), 2541–2548. 10.1099/ijs.0.2008/000893-0 18984690

[B63] Senko J. M. Zhang G. McDonough J. T. Bruns M. A. Burgos W. D. (2009). Metal Reduction at Low pH by aDesulfosporosinusspecies: Implications for the Biological Treatment of Acidic Mine Drainage. Geomicrobiol. J. 26 (2), 71–82. 10.1080/01490450802660193

[B64] Sieber C. M. K. Probst A. J. Sharrar A. Thomas B. C. Hess M. Tringe S. G. (2018). Recovery of Genomes from Metagenomes via a Dereplication, Aggregation and Scoring Strategy. Nat. Microbiol., 3:836–843. 10.1038/s41564-018-0171-1 29807988 PMC6786971

[B65] Stamatakis A. (2014). RAxML Version 8: a Tool for Phylogenetic Analysis and Post-analysis of Large Phylogenies. Bioinformatics 30 (9), 1312–1313. 10.1093/bioinformatics/btu033 24451623 PMC3998144

[B66] Stockdreher Y. Venceslau S. S. Josten M. Sahl H.-G. Pereira I. A. C. Dahl C. (2012). Cytoplasmic Sulfurtransferases in the Purple Sulfur Bacterium Allochromatium Vinosum: Evidence for Sulfur Transfer from DsrEFH to DsrC. PLoS One 7 (7), e40785. 10.1371/journal.pone.0040785 22815818 PMC3397948

[B67] Van der Graaf C. M. Sánchez-España J. Yusta I. Ilin A. Shetty S. A. Bale N. J. (2020). Biosulfidogenesis Mediates Natural Attenuation in Acidic Mine Pit Lakes. Microorganisms 8 (9), 1275. 10.3390/microorganisms8091275 PMC7565709 32825668

[B68] Venceslau S. S. Stockdreher Y. Dahl C. Pereira I. A. C. (2014). The “Bacterial Heterodisulfide” DsrC Is a Key Protein in Dissimilatory Sulfur Metabolism. Biochimica Biophysica Acta (BBA) - Bioenergetics 1837 (7), 1148–1164. 10.1016/j.bbabio.2014.03.007 24662917

[B69] Vigneron A. Cruaud P. Culley A. I. Couture R.-M. Lovejoy C. Vincent W. F. (2021). Genomic Evidence for Sulfur Intermediates as New Biogeochemical Hubs in a Model Aquatic Microbial Ecosystem. Microbiome 9 (1), 46. 10.1186/s40168-021-00999-x 33593438 PMC7887784

[B70] von Meijenfeldt F. A. B. Arkhipova K. Cambuy D. D. Coutinho F. H. Dutilh B. E. (2019). Robust Taxonomic Classification of Uncharted Microbial Sequences and Bins with CAT and BAT. Genome Biol. 20 (1), 217. 10.1186/s13059-019-1817-x 31640809 PMC6805573

[B71] Wasmund K. Cooper M. Schreiber L. Lloyd K. G. Baker B. J. Petersen D. G. (2016). Single-Cell Genome and Group-Specific dsrAB Sequencing Implicate Marine Members of the Class Dehalococcoidia (Phylum Chloroflexi) in Sulfur Cycling. mBio 7 (3), e00266–16. 10.1128/mbio.00266-16 27143384 PMC4959651

[B72] Wendt-Potthoff K. Koschorreck M. Diez Ercilla M. Sánchez España J. (2012). Microbial Activity and Biogeochemical Cycling in a Nutrient-Rich Meromictic Acid Pit Lake. Limnologica 42 (3), 175–188. 10.1016/j.limno.2011.10.004

[B73] Willis G. Nancucheo I. Hedrich S. Giaveno A. Donati E. Johnson D. B. (2019). Enrichment and Isolation of Acid-Tolerant Sulfate-Reducing Microorganisms in the Anoxic, Acidic Hot Spring Sediments from Copahue Volcano, Argentina. FEMS Microbiol. Ecol. 95 (12). fiz175. 10.1093/femsec/fiz175 31665270

[B74] Wolff D. G. Bidlack W. R. (1976). The Formation of Carbon Monoxide during Peroxidation of Microsomal Lipids. Biochem. Biophysical Res. Commun. 73 (4), 850–857. 10.1016/0006-291x(76)90199-6 15625852

[B75] Wrighton K. C. Castelle C. J. Varaljay V. A. Satagopan S. Brown C. T. Wilkins M. J. (2016). RubisCO of a Nucleoside Pathway Known from Archaea Is Found in Diverse Uncultivated Phyla in Bacteria. Isme J. 10 (11), 2702–2714. 10.1038/ismej.2016.53 27137126 PMC5113843

[B76] Wu Y.-W. Simmons B. A. Singer S. W. (2016). MaxBin 2.0: an Automated Binning Algorithm to Recover Genomes from Multiple Metagenomic Datasets. Bioinformatics 32 (4), 605–607. 10.1093/bioinformatics/btv638 26515820

[B77] Zhou Z. Tran P. Q. Breister A. M. Liu Y. Kieft K. Cowley E. S. (2020). METABOLIC: High-Throughput Profiling of Microbial Genomes for Functional Traits, Biogeochemistry, and Community-Scale Metabolic Networks. bioRxiv 10, 761643. 10.1186/s40168-021-01213-8 PMC8851854 35172890

