# Supplementary figures and images for: Novel Microorganisms Contribute to Biosulfidogenesis in the Deep Layer of an Acidic Pit Lake
Source: Front Bioeng Biotechnol. 2022 Jul 13;10:867321. doi: 10.3389/fbioe.2022.867321 (PMC9326234; doi:10.3389/fbioe.2022.867321)

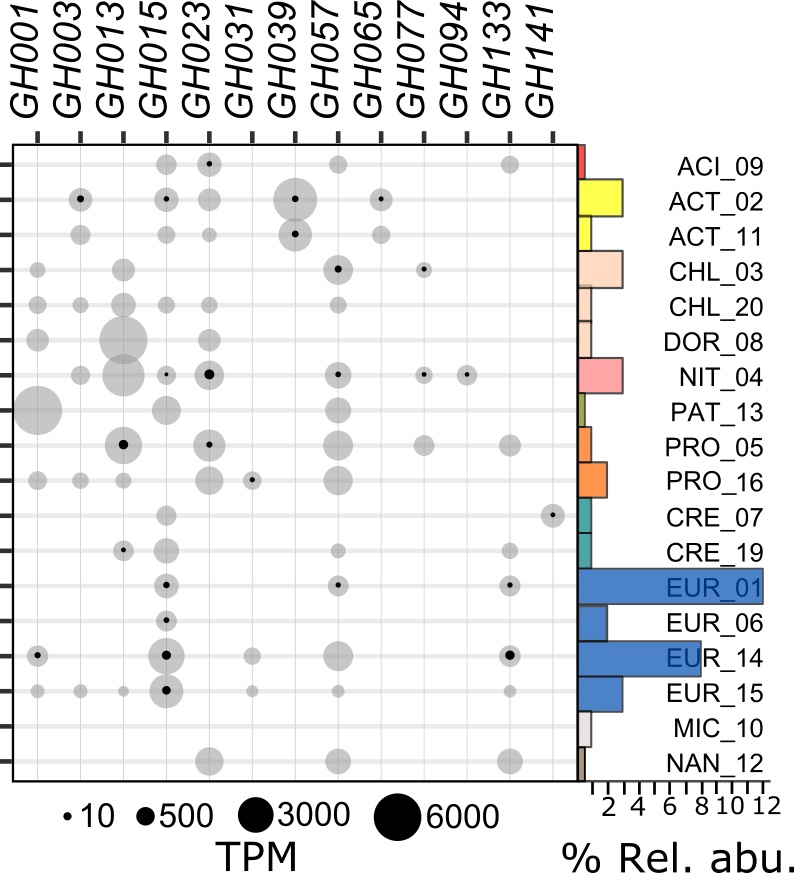

Supplement: Supplementary file 1 [file Image1.jpeg]

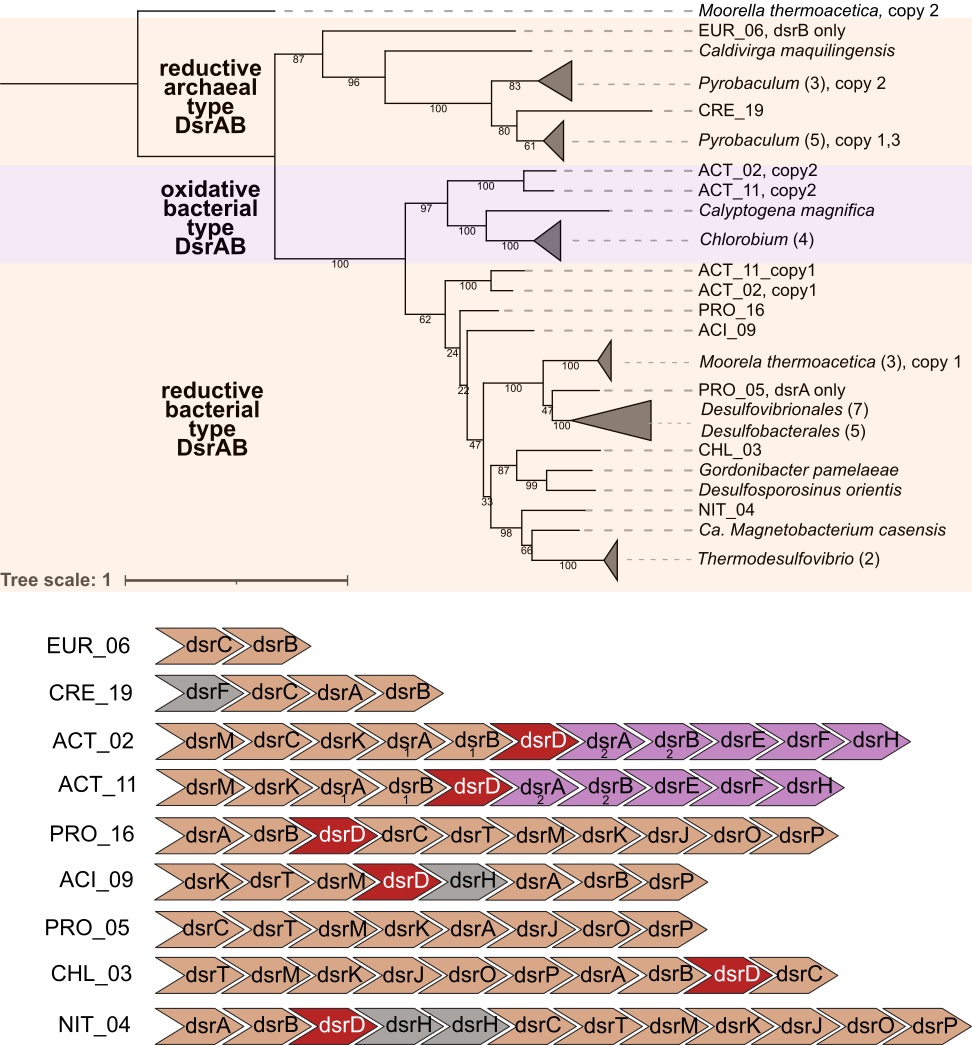

Supplement: Supplementary file 2 [file Image2.jpeg]
